# Single-cell transcriptomics reveals heterogeneous neutrophil populations and diagnostic biomarkers in atherosclerosis

**DOI:** 10.3389/fphys.2026.1704443

**Published:** 2026-03-02

**Authors:** Ping Wang, Qiang Fu, Yuefeng Cai, Jiaxin Yang, Zhenzhen Cui, Qiyu Sun, Quanli Qiu, Xiaowen Ma, Min Li

**Affiliations:** 1 Department of Nuclear Medicine, The 960th Hospital of the PLA, Jinan, Shandong, China; 2 Medical Service Department, The 960th Hospital of the PLA, Jinan, Shandong, China; 3 College of Liberal Arts and Sciences, University of Illinois Urbana-Champaign, Champaign, IL, United States; 4 Jinzhou Medical University Graduate Training Base (The 960th Hospital of the PLA), Jinan, Shandong, China; 5 Department of Pharmacy, The 960th Hospital of the PLA, Jinan, Shandong, China

**Keywords:** atherosclerosis, biomarkers, immune cells, neutrophil subtypes, single-cell RNA sequencing

## Abstract

**Introduction:**

Atherosclerosis, a chronic inflammatory arterial disease, involves complex interactions among diverse immune cells. Although single-cell RNA sequencing (scRNA-seq) has revealed cellular heterogeneity within atherosclerotic lesions, the diversity of neutrophils and their crucial genes in atherogenesis remain elusive.

**Methods:**

We integrated scRNA-seq data from six human atherosclerotic samples, comprising 47,604 single cells.The Harmony algorithm was applied for dimensionality reduction and batch effect correction, resulting in the identification of 16 distinct cell subtypes. High-resolution weighted gene co-expression network (hdWGCNA) analysis was performed to construct a gene co-expression network specifically within the neutrophil subtype. Characteristic genes were selected using Lasso regression. Immune infiltration analysis, gene set enrichment analysis (GSEA), and gene set variation analysis (GSVA) were conducted to investigate the biological functions and potential regulatory mechanisms of the key genes. An atherosclerosis animal model was employed to validate the expression levels of the key genes in carotid artery tissues. In addition, transcriptional regulatory network analysis and correlation analysis between key genes and atherosclerosis-related regulatory genes were carried out. Drug prediction was performed using the Connectivity Map (CMap) database.

**Results:**

A total of 16 cell subtypes were identified following batch effect correction. Annotation results revealed a increased proportion of neutrophils in atherosclerotic arteries compared to healthy controls. WGCNA analysis of neutrophil subtypes unveiled four key genes: CSTB, CHST15, RNASE1 and ATP2B1, which were validated as promising candidate biomarkers in independent cohorts (AUC >0.85). Preliminary validation in the animal model showed that the expression levels of the four genes were elevated in diseased carotid artery tissues compared to normal controls. Immune infiltration analysis demonstrated significant correlations between the key genes and the infiltration levels of multiple immune cell types. GSEA and GSVA showed that these genes were primarily enriched in pathways associated with inflammatory response, ferroptosis, and atherosclerosis-related signaling pathways. Transcriptional regulation analysis predicted potential transcription factors, and correlation analysis revealed significant associations between the key genes and known atherosclerosis-related regulatory genes. CMap analysis predicted several small-molecule compounds with potential therapeutic effects.

**Discussion:**

Our findings uncover the previously unrecognized heterogeneity of neutrophils in atherosclerosis and identify four key genes as promising diagnostic markers and therapeutic targets. These findings provide new insights into the pathogenesis of atherosclerosis and provide a theoretical basis for developing targeted therapies against this devastating disease.

## 1 Introduction

Atherosclerosis (AS) is characterized as a chronic inflammatory disease affecting the arterial wall, is a leading cause of global mortality, underlying numerous cardiovascular disorders such as coronary heart disease, stroke, and peripheral arterial disease (Roth et al., 2020). The progression of atherosclerosis involves endothelial dysfunction, oxidized low-density lipoprotein (ox-LDL) deposition, and a complex interplay of various immune cells, including macrophages, dendritic cells, neutrophils, T cells, and B cells (Wojtasińska et al., 2023). These cells contribute to plaque formation and instability through cytokine and chemokine secretion (Peter Libby, 2013).

Recent studies have identified potential immunotherapy targets for atherosclerosis, such as inhibitors of inflammatory factors and chemokines (Meng et al., 2023). However, the efficacy of these interventions may depend on the heterogeneous composition and functional states of immune cells within atherosclerotic lesions. Moreover, immune cell interactions, such as neutrophil-macrophage crosstalk mediated by neutrophil extracellular traps (NETs) and protease 3, have been shown to promote inflammation, plaque formation, and instability (Soehnlein et al., 2017). Yet, the impact of these cellular networks on plaque vulnerability and the underlying molecular mechanisms remain incompletely understood.

The advent of scRNA-seq has provided a powerful tool to address the heterogeneity of immune cells in atherosclerotic lesions. By revealing distinct cell subpopulations and their unique gene expression profiles, scRNA-seq studies have identified specific macrophage and T cell subsets associated with atherosclerosis progression and prognosis (Li et al., 2022; Cochain et al., 2018; Xiong et al., 2023). These findings underscore the immense potential of scRNA-seq technology in uncovering novel insights into the diversity and roles of immune cells in atherosclerosis. Despite the increasing application of single-cell sequencing in atherosclerosis research, the heterogeneity of neutrophils and their roles in disease progression remain unexplored. Neutrophils are among the most abundant leukocytes in atherosclerotic plaques and contribute to disease initiation and progression through various mechanisms, such as releasing inflammatory mediators, reactive oxygen species, and proteolytic enzymes (Carlos Silvestre-Roig and Christian Weber, 2019; Yvonne Döring, 2020). There is an urgent need to construct a comprehensive cellular atlas of atherosclerosis based on multi-omics integration, identify disease-specific key driver genes and pathways, and develop targeted diagnostic and therapeutic strategies (Depuydt et al., 2020).

To fill the knowledge gaps in understanding the cellular heterogeneity and molecular mechanisms underlying atherosclerosis progression, we integrated multiple single-cell transcriptome datasets comprising 47,604 immune cells derived from six human atherosclerotic samples. Specifically, we aimed to: (1) characterize the immune cell landscape and identify cell subpopulations with altered proportions in atherosclerotic lesions; (2) construct gene co-expression networks and identify key genes within specific immune cell subtypes, particularly neutrophils; (3) investigate the relationships between the key genes and various immune cells and factors; and (4) explore the potential molecular mechanisms through which these key genes influence atherosclerosis progression. By leveraging the power of scRNA-seq technology and bioinformatic analyses, these characteristic genes could prove to be crucial regulators and potential therapeutic targets in atherosclerosis.

## 2 Materials and methods

### 2.1 Data set source

This study utilized three public datasets: the scRNA-seq dataset GSE159677 and two bulk microarray datasets (GSE28829 and GSE43292).

### 2.2 Data acquisition from gene expression omnibus database

To conduct our bioinformatics analysis, we obtained gene expression data from the Gene Expression Omnibus (GEO, http://www.ncbi.nlm.nih.gov/geo/) (Barrett et al., 2013).

GSE159677: Single-cell analysis was performed on a total of six samples obtained from three patients undergoing carotid endarterectomy. These comprised three calcified atherosclerotic core (AC) plaques and three patient-matched proximal adjacent (PC).

GSE43292: The Series Matrix File was downloaded and annotated using the corresponding GPL6244 (Affymetrix Human Gene 1.0 ST Array) platform file. This dataset was generated from paired tissue samples obtained *via* carotid endarterectomy from 32 hypertensive patients. For each patient, two tissue types were collected: atheroma plaque (ATH), representing the diseased tissue (n = 32), and macroscopically intact tissue (MIT) from a proximal adjacent region, serving as a patient-matched internal control (n = 32).

GSE28829: The Series Matrix File for this dataset was downloaded and annotated using the GPL570 platform file. It included expression data from 29 patients, categorized into early atherosclerosis from asymptomatic patients with carotid artery stenosis (n = 13) and advanced atherosclerosis from patients with symptomatic carotid artery stenosis (n = 16).

### 2.3 Single-cell RNA-seq data analysis

ScRNA-seq data were processed using the Seurat package. Low-quality cells and genes were filtered by applying thresholds (nFeature_RNA >200, nFeature_RNA <4,000, and percent. mt < 15). The data were then log-normalized, scaled, and subjected to principal component analysis (PCA). The optimal number of principal components was selected based on the ElbowPlot. Dimensionality reduction and visualization were performed using Uniform Manifold Approximation and Projection (UMAP) to reveal the spatial relationships among clusters. Cell clusters were annotated according to well-established marker genes from the literature and the CellMarker database (Zhang et al., 2019), and were assigned to arterial tissue–relevant cell types.

### 2.4 Intercellular communication network analysis

To systematically map the cellular crosstalk within the atherosclerotic microenvironment, we performed a quantitative analysis of intercellular communication networks using CellChat (https://github.com/sqjin/CellChat) (Jin et al., 2021). Standardized single-cell expression profiles served as the input. Cell subtypes identified through single-cell clustering were annotated based on established marker genes. Cellular interactions were systematically analyzed, with interaction strength quantified using both interaction weights and interaction counts between cell types. This network-based approach enables the assessment of cellular activity and functional influence within the disease microenvironment (Xu et al., 2025).

### 2.5 Gene co-expression network analysis

Gene co-expression networks were constructed using high-dimensional Weighted Gene Co-expression Network Analysis (hdWGCNA) (Morabito et al., 2023). We initiated our analysis by constructing a co-expression network using the SetupForWGCNA function. This network encompassed genes expressed in at least 5% of cells within our Seurat objects, ensuring a comprehensive yet focused analysis. We applied a soft threshold of nine to optimize the network’s scale-free topology. The resulting network structure was visualized using the PlotDendrogram function, which produces a hierarchical clustering tree. In this visualization, each leaf of the tree represents an individual gene, while the colors at the base of the tree denote the allocation of genes to distinct co-expression modules. Finally, the MEs level of each module is visualized by GetMEs.

### 2.6 Predictive model development for atherosclerosis risk

Select candidate genes and further construct a predictive model using lasso regression. After including the expression values of each specific gene, a risk score formula was constructed for each patient, and the estimated regression coefficients from the lasso regression analysis were weighted for use in the model. Based on the risk score formula, the patients were divided into low-risk and high-risk groups using the median risk score value as the dividing point, and the accuracy of the model’s prediction was studied using the ROC curve (Xu et al., 2022).

### 2.7 Immune cell infiltration analysis

CIBERSORT method (https://cibersort.stanford.edu/) (Newman et al., 2015) is a widely used method to evaluate immune cell types in microenvironment. Based on the principle of support vector regression, the expression matrix of immune cell subtypes was deconvolution analyzed. It contains 547 biomarkers that distinguish 22 human immune cell phenotypes, including T cells, B cells, plasma cells, and myeloid cell subsets. In this study, CIBERSORT algorithm was used to analyze patient data to infer the relative proportions of 22 types of immune infiltrating cells.

### 2.8 Gene set enrichment analysis

The patients were divided into high and low expression groups according to the high and low expression of genes, and the difference of signaling pathway between the two groups was further analyzed by GSEA. Background gene sets were annotated gene sets downloaded from MsigDB database (http://software.broadinstitute.org/gsea/msigdb) as annotated gene sets of subtype pathways. Differential expression analysis of pathways between subtypes was performed, and significantly enriched gene sets (adjusted p value less than 0.05) were sorted according to consistency scores.

### 2.9 Regulatory network analysis of key genes

In this study, the R package “RcisTarget” (Aibar et al., 2017) was used to predict transcription factors. All analyses performed by RcisTarget are fundamentally based on DNA sequence motifs. The normalized enrichment score (NES) for each motif was calculated, taking into account the total number of motifs in the reference database. In addition to the motifs directly annotated in the source data, we inferred further annotations based on motif similarity and gene sequences. The first step in estimating the overexpression of each motif on the gene set is to calculate the area under the curve (AUC) of each pair of motif-motif sets. This is calculated by the recovery curve of the sequence based on the gene set. The NES for each motif was then derived from the distribution of AUC values across all motifs in the gene set.

### 2.10 CMap drug prediction

We conducted an analysis of Atherosclerosis drug reuse using the Connectivity Map (CMap, version 2.0) (Wang et al., 2025). A query gene signature was constructed from the GSE43292 dataset. Differentially expressed mRNAs between Atherosclerosis and control samples were identified using the limma package in R. The top 150 upregulated and the top 150 downregulated genes (ranked by adjusted p-value and log_2_ fold change) were selected to form the Atherosclerosis -associated signature. This signature was then submitted to the CMap web portal (https://clue.io/) and analyzed using the built-in pattern-matching algorithm (XSum). Compounds with significantly negative connectivity scores (indicating a reversal of the disease signature) were considered candidate therapeutic agents and prioritized for further investigation. The analysis threshold was set at |connectivity score| >90 and p-value <0.05.

### 2.11 Animal models for atherosclerosis

The use of animals and the study protocols were approved by the Scientific Research Ethics Committee of PLA 960th Hospital.

To establish atherosclerosis models, we employed both mice and rats. For the mouse model, we used six male C57BL/6J mice and six male ApoE^−/−^ mice, all 6–8 weeks old. The C57BL/6J mice were fed a normal diet, while the ApoE^−/−^ mice received a high-fat diet (HFD). For the rat model, we used twelve 6-week-old male Sprague-Dawley (SD) rats, randomly divided into control and atherosclerosis groups. In the atherosclerosis group, the left common carotid artery was exposed and clamped with a microvascular clip for 30–40 min to induce endothelial injury *via* ischemia-reperfusion. On the first postoperative day, these rats received an intraperitoneal injection of vitamin D3 at a dose of 600,000 IU/kg, followed by additional vitamin D3 injections (100,000 IU/kg) at weeks 3, 6, and 9 after surgery. Throughout the 12-week experimental period, rats in the atherosclerosis group were fed a high-fat diet to promote hyperlipidemia and plaque formation. Rats in the control group underwent a sham operation (vessel exposure without clamping) and were maintained on standard chow. The high-fat diet consisted of 10% lard, 3% cholesterol, 0.5% cholic acid, 0.2% propylthiouracil, and 86.3% basic feed.

### 2.12 Histological examination

Mice were anesthetized intraperitoneally before surgical removal of the heart and aortic arch, from the proximal aortic root to the iliac artery branch. This tissue was immersed in fixative solution. The following day, the aorta was carefully separated, surrounding adipose tissue was removed, and it was again immersed in fixative. Oil Red O staining kit (Beyotime C0157S) was used for gross staining, with subsequent quantitative analysis of plaques performed using ImageJ software.

Rat liver tissues were preserved in fixative solution. A portion of the liver tissue was sectioned into 4 μm slices, dehydrated, embedded in paraffin, and prepared for hematoxylin and eosin (HE) staining.

### 2.13 Blood fats examination

After 3 months of high-fat diet, serum samples were collected from each group by centrifugation at 2000 rpm for 15 min at 4 °C. The serum of mice and rats was placed in dry ice and transported to Wuhan Sibe Bio Company, and total cholesterol (TC), triglycerides (TG), low-density lipoprotein cholesterol (LDL-C), and high-density lipoprotein cholesterol (HDL-C) were detected by biochemical analyzer.

### 2.14 3DcsTof magnetic resonance imaging (MRI)

MRI scans were conducted 48 h after rat modeling using a Ingenia 3.0T MRI scanner (Philips) equipped with a 16-channel longitudinal rat coil (Shanghai Chenguang Medical Technologies Co., Ltd.). Prior to scanning, rats were anesthetized with isoflurane and positioned prone on the scanning platform. The central point of the brain was aligned perpendicular to the baseline scan, using the line connecting the forebrain tip to the central point as a reference.

3D Arterial Spin Labeling (3D-ASL):

TR/TE: 23 m/2.9 ms

3D spiral fast spin echo with eight spiral arms

512 points acquired per arm

Post-labeling delay time: 1,000 ms

Number of excitations (NEX): 3

Slice thickness: 2 mm

FOV = 60 mm × 60 mm

### 2.15 Western blotting analysis

Artery tissues from mice or rats were homogenized in 200 μL RIPA lysis solution. The tissue lysates were mixed with 5x loading buffer and boiled for 10 min at 100 °C. Proteins were then separated by electrophoresis on 10% sodium dodecyl sulfate-polyacrylamide gels and transferred to nitrocellulose membranes. The membranes were blocked with 5% skimmed milk in Tris-buffered saline containing 0.1% Tween 20 (TBST) for 1 h at room temperature. Primary antibodies were applied and incubated overnight at 4 °C. These included rabbit anti-ATP2B1 (1:1,000 dilution, Proteintech 30035-1-AP), rabbit anti-CHST15 (1:1,000 dilution, Proteintech 14298-1-AP), rabbit anti-CSTB (1:1,000 dilution, Fisher Scientific PIPA566210), and mouse anti-actin (1:1,000 dilution, ZSGB-BIO TA-09). Following incubation, the membranes were washed three times with TBST and then incubated with appropriate anti-mouse or anti-rabbit secondary antibodies for 1 h. Immunoreactive bands were visualized using the SuperSignal West Pico PLUS chemiluminescent substrate (Thermo Fisher Scientific) and imaged on the Tanon-4800 Multi chemiluminescence imaging system. Band intensity was quantified using ImageJ. To ensure reliability, all protein expression experiments were independently repeated three times.

### 2.16 Quantitative PCR analysis

Aortic tissue from the control and disease groups of mice or rats was used for RNA extraction using an RNA tissue extraction kit. Total RNA was reverse-transcribed into cDNA using the PrimeScript RT reagent Kit with gDNA Eraser (TaKaRa, Japan). mRNA expression was quantified using a SLAN-96P real-time PCR system. Actin served as the endogenous reference gene, and the 2^−ΔΔCT^ method was employed to normalize mRNA expression levels between groups. All samples were tested in triplicate. Primers were obtained from Sangon Biotech, with sequences as follows: ATP2B1: Forward: 5′-ACCATTCCAACCAGCCGCTTAAAG-3′ Reverse: 5′-ATCCTCCGCCAATTCCTCCTCAG-3′ CSTB: Forward: 5′-CGCCATCCGCCACAATGCC-3′ Reverse: 5′-CCGGCCACTACCTGTCTCCTG-3′ CHST15: Forward: 5′-TGCGTCTACAACAACAACCCTGACC-3′ Reverse: 5′-ACAGTGAGCCAGTCCAGGAGATAC-3′ RNASE1: Forward: 5′-CATCACTGACTGCCGCCTGAAG-3′ Reverse: 5′- CACGGAAGCATCGAAGTGGACTG-3′.

### 2.17 Statistical analysis

Bioinformatic analyses were conducted using R (version 4.3.2). Statistical analysis of experimental data was performed with SPSS (version 27.0) (Ma et al., 2025). Data visualization was created using GraphPad Prism (version 9.0). Experimental data are presented as mean ± standard deviation (SD). Comparisons between two groups were analyzed using an unpaired, two-tailed Student’s t-test. Comparisons among multiple groups were performed by one-way analysis of variance. *P* < 0.05 was considered statistically significant.

## 3 Results

### 3.1 Pre-processing of single cell expression profile data and annotation of cell subsets

**FIGURE 1:**
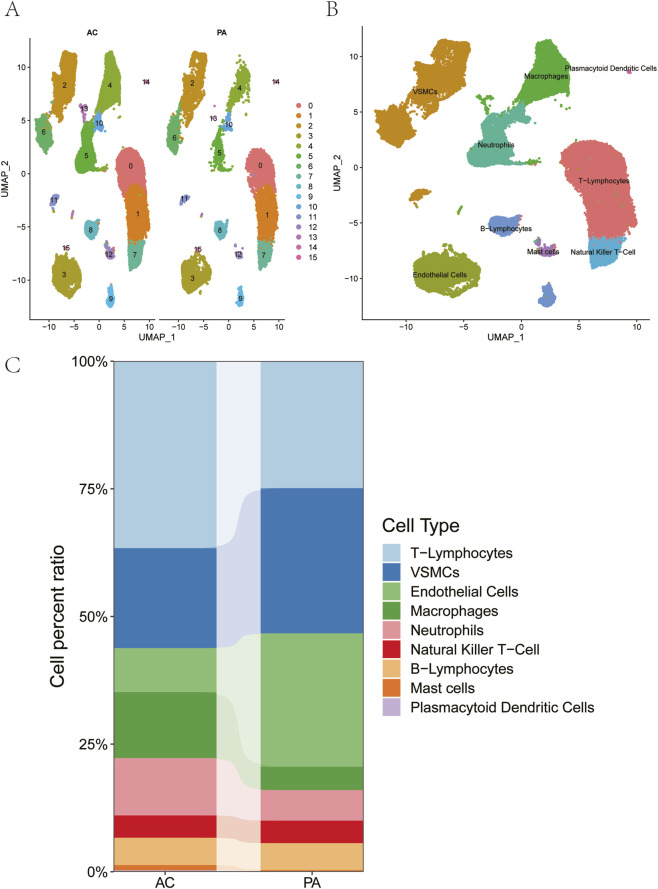
Cluster analysis of cell subtypes. **(A)** UMAP visualization showing the overall cell clustering in AC and PA samples. **(B)** Sub-clustering analysis of specific cellular populations within AC samples. **(C)** The percentage distribution of different cell subtypes in AC and PA samples.

This analysis was performed on expression profiles from six samples relevant to atherosclerosis. Only cells with nFeature_RNA >200 and nFeature_RNA <4,000 and percent. mt < 15 in the expression profile were retained for this analysis. The feature expression levels of a total of 47,604 cells were included for subsequent analysis (Supplementary Figure 1A,B). The 10 genes with the highest standard deviation were shown (Supplementary Figure 1C). In this study, it was found that batch effect existed among samples through PCA dimensionality reduction analysis (Supplementary Figure 1D), and Harmony analysis was further used to reduce dimensionality and batch removal (Supplementary Figure 1E). The optimal number of PCs, determined as 14 by the ElbowPlot (Supplementary Figure 1F), UMAP was then performed, revealing 16 distinct cell subtypes (Figure 1A).

### 3.2 Annotation of cluster subtypes and analysis of receptor-ligand relationship pairs

**FIGURE 2:**
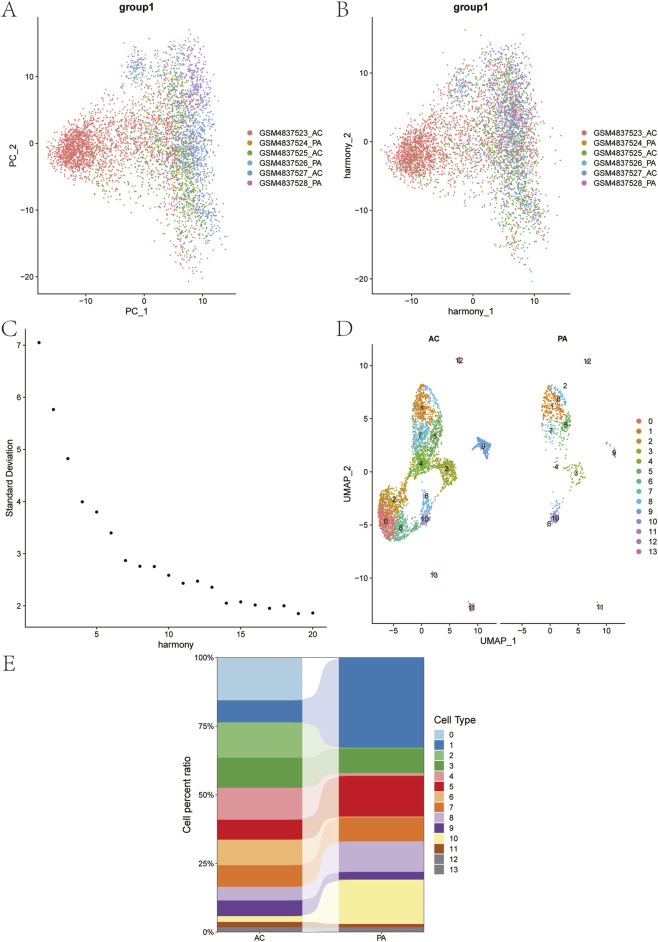
Cluster analysis of neutrophil subtypes. **(A–D)** Pre-processing of cell expression profile data. **(A)** PCA dimensionality reduction analysis; **(B)** Harmony dimensionality reduction analysis; **(C)** The optimal number of PCs; **(D)** Clustering UMAP plots of different clusters of neutrophils in AC and PA samples. **(E)** The percentage distribution of different neutrophil subtypes in AC and PA samples.

**FIGURE 3:**
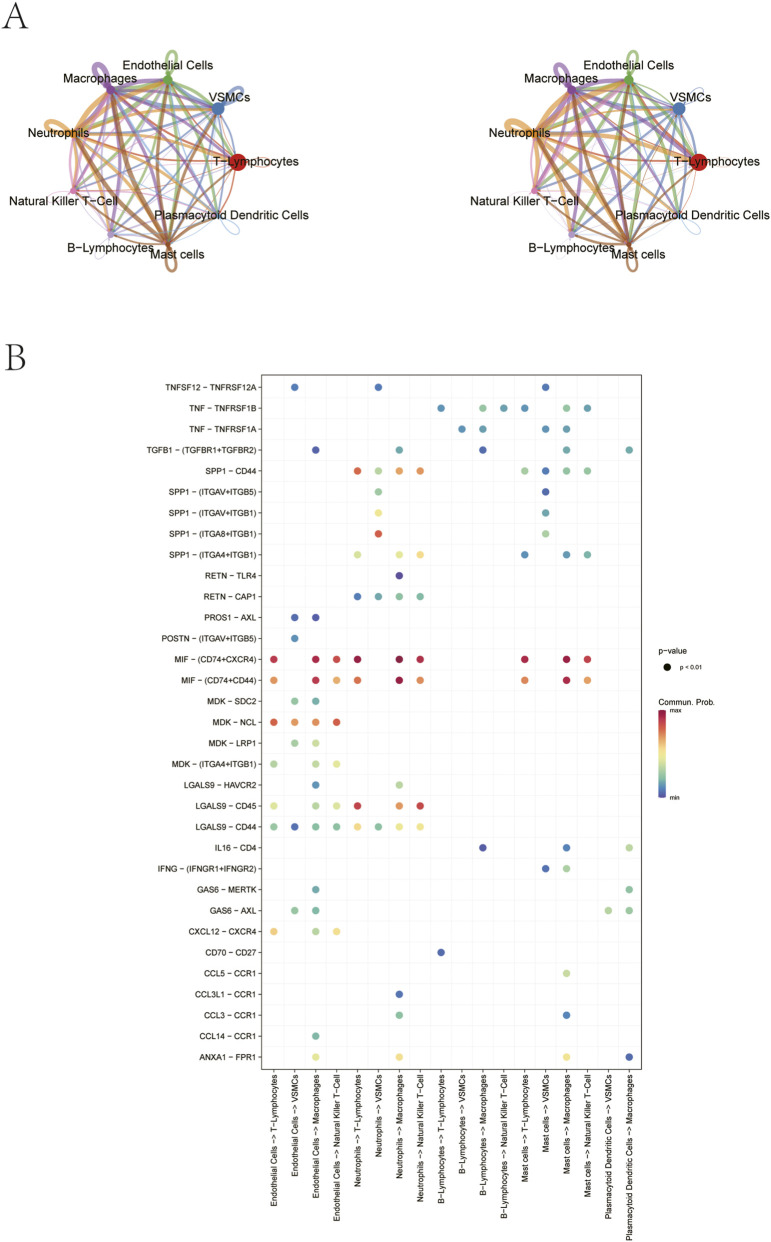
CellChat analysis of intercellular communication using scRNA-seq data. **(A)** Cellular interactions network plot. **(B)** Analysis of receptor-ligand relationship pairs.

Based on canonical marker genes referenced from the literature and the CellMarker database, the 16 clusters were annotated and grouped into nine major cell categories: T-Lymphocytes, VSMCs, Endothelial Cells, Macrophages, Neutrophils, Natural Killer T-Cells, B-Lymphocytes, Mast Cells, and Plasmacytoid Dendritic Cells (Figure 1B). Importantly, the proportion of neutrophils was significantly higher in the AC group than in the PA group (Figure 1C). Further subsets of Neutrophils subtypes were analyzed by PCA, Harmony, ElbowPlot and FindClusters successively (Figures 2A–D). Among them, the proportions of C0, C2, C4, C6, C9, and C11 were significantly increased in AC group compared to PA group (Figure 2E). In this study, the ligand-receptor relationship of Fature in single cell expression profile was analyzed by cellchat software package. Complex interaction pairs were found among these cell subtypes (Figure 3A, B).

### 3.3 hdWGCNA analysis

**FIGURE 4:**
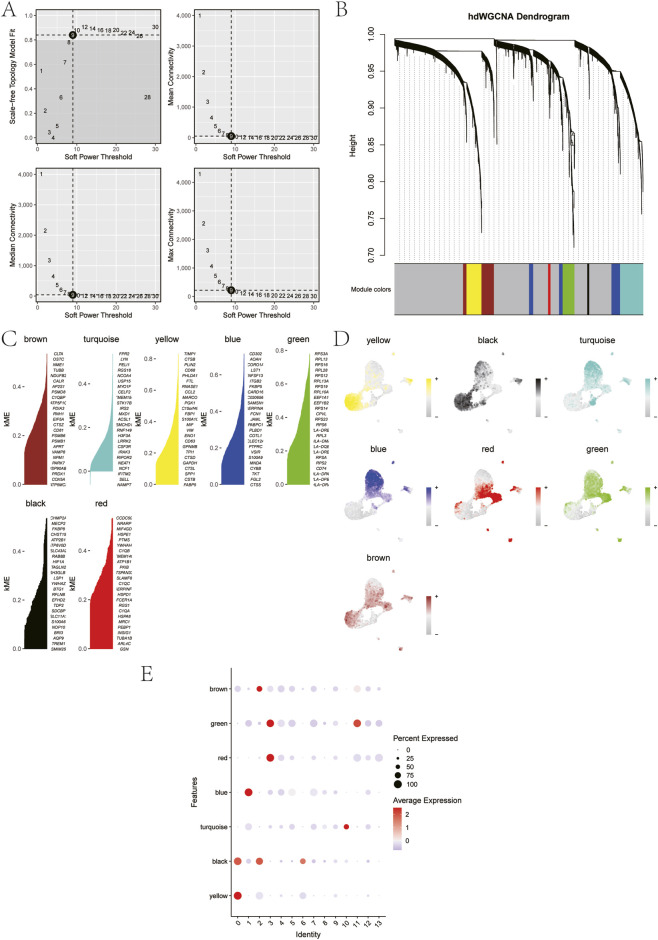
hdWGCNA analysis of neutrophil subtypes. **(A)** Optimal soft threshold selection. **(B)** Construction of co-expression network using the optimal soft threshold of 9, with genes divided into seven modules and resulting in a dendrogram. **(C)** Distribution of module membership (kME) values for genes across all modules. **(D)** UMAP visualization of neutrophil clusters, colored by module eigengene (ME) expression. **(E)** Module activity for 13 neutrophils clusters.

To construct a gene co-expression network specific to neutrophil subtypes, we performed hdWGCNA analysis using “group.by” parameters defined by cluster and custom subtypes. This network was subsequently utilized to identify key biomarkers associated with atherosclerotic progression. The soft threshold is determined by the function “TestSoftPowers” and is set to 9 (Figure 4A). A total of seven gene modules were detected in this analysis, which were successively brown, turquoise, yellow, blue, green, black and red modules (Figure 4B). Further correlation analysis between gene co-expression modules and their MEs (Figures 4C,D) revealed that the black and yellow modules exhibited significantly elevated MEs levels specifically within the atherosclerotic-associated cell subtypes (Figure 4E). The top 25 genes were extracted from the black and yellow modules respectively as candidate genes for subsequent research.

### 3.4 Lasso regression and SVM feature selection

**FIGURE 5:**
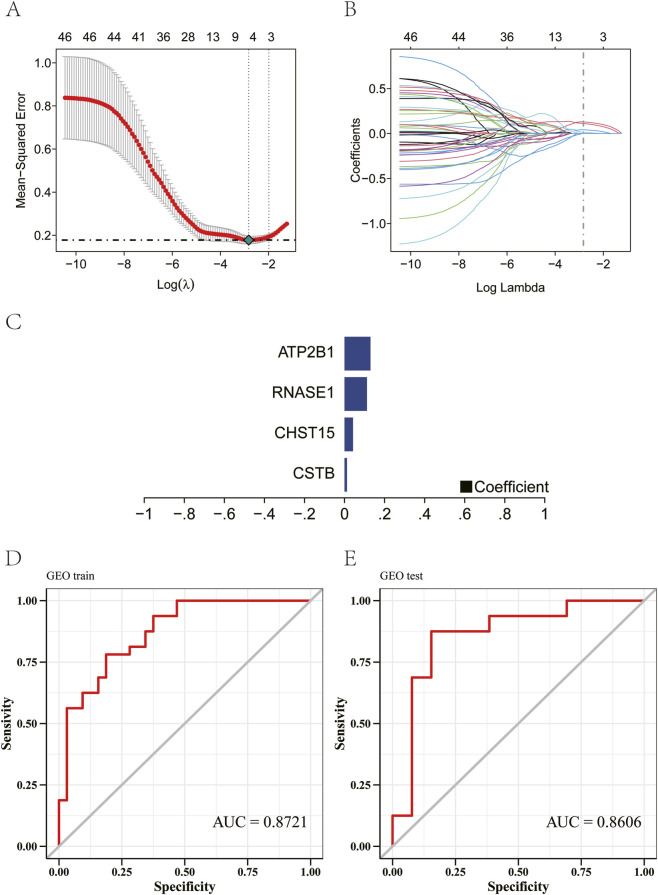
Screening of atherosclerotic characteristic genes. **(A, B)** The predictors were selected using LASSO regression. **(C)** Characteristic genes screened by LASSO regression. **(D)** The ROC curve verifies the accuracy of the model prediction.

To identify the core molecular features of atherosclerosis, we used the GSE43292 dataset as a training set and performed feature selection on candidate genes obtained from prior analyses using Lasso regression. The results showed that Lasso regression consensus identified four genes as characteristic genes of atherosclerosis (Figures 5A–C), and the model formula was as follows: RiskScore = CSTB × 0.0122319817272104 + CHST15 × 0.0424346201532444 + RNASE1 × 0.112072455005877 + ATP2B1 × 0.129722204301299. Based on these four characteristic genes, we constructed a classification model for disease risk prediction. The GSE28829 dataset was employed as an independent external validation set to evaluate the model. The results showed that the four-gene predictive model demonstrated robust diagnostic performance in distinguishing high-risk (symptomatic) from low-risk (asymptomatic) atherosclerotic plaques, and the area under the AUC curve was 0.8721 (Figure 5D). Furthermore, when rigorously validated using the GSE28829 dataset as an external test set, the model consistently maintained robust and stable performance, with an area under the AUC curve of 0.8606 (Figure 5E), confirming its generalizability and reliability for clinical risk stratification.

### 3.5 The expression of key genes was verified in rat and mouse arterial tissues

**FIGURE 6:**
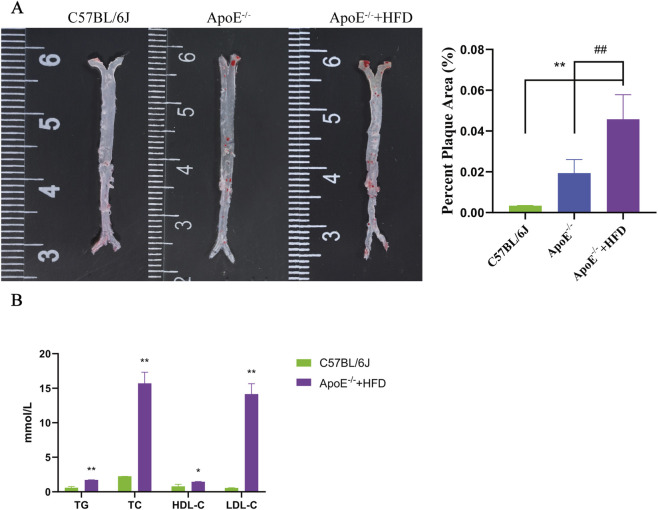
Validation of atherosclerosis model in mice. **(A)** Representative images of oil red O staining of mouse aorta are shown on the left panel, and statistical graphs are shown on the right panel indicating the quantification of aortic plaque area in each group (n = 5). **(B)** Effects of a high-fat diet on blood lipids in ApoE^−/−^ mice (n = 5). Error bars represent standard deviation (*P < 0.05, **P < 0.01, compared with the C57BL/6J mice group, ^##^ *P* < 0.01, compared with ApoE^−/−^ mice group, LSD t-test).

**FIGURE 7:**
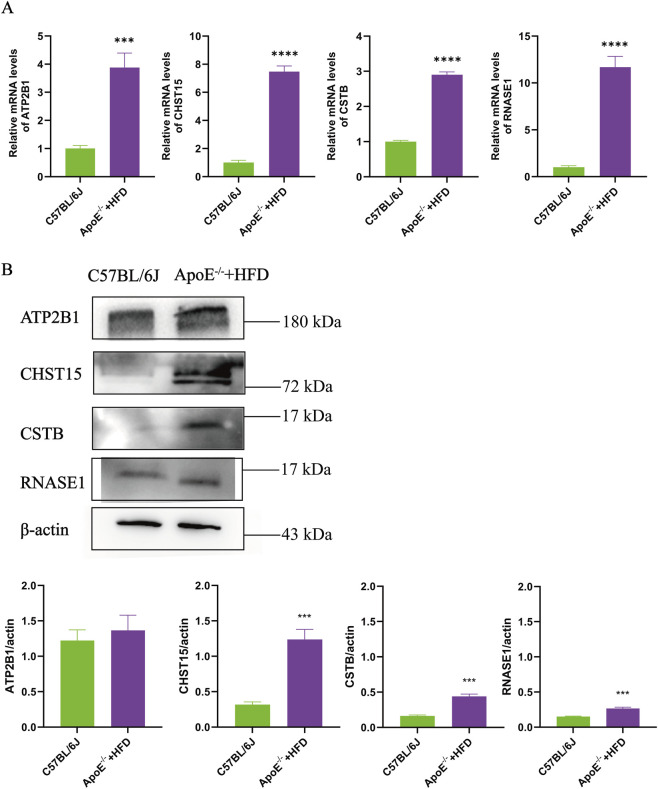
Expression levels of ATP2B1, CHST15, CSTB and RNASE1 in mouse arteries. **(A)** The gene levels of ATP2B1, CHST15, CSTB and RNASE1 were analyzed by Real-time PCR. **(B)** The protein levels of ATP2B1, CHST15, CSTB and RNASE1 were analyzed by western blot. Actin was used as a loading control. Data represent mean ± standard deviation of three independent experiments (***P < 0.001, ****P < 0.0001, compared with the C57BL/6J mice, two-tailed Student’s t-test).

6-week-old ApoE^−/−^ mice were fed with high-fat diet for 4 months to establish advanced atherosclerotic lesions. Whole aorta oil red O staining showed that the vascular plaque area of ApoE^−/−^ mice on high-fat diet was 8.6% larger than that of C57BL/6J mice, and 6.1% larger than that of ApoE^−/−^ mice on normal diet (Figure 6A). As shown in Figure 6B, serum levels of TG, TC and LDL-C in the ApoE^−/−^ +HFD group were significantly higher than those in the C57BL/6J group (*P* < 0.01). The results showed that high-fat diet potently aggravates atherosclerosis in ApoE^−/−^ mice. Real-time PCR and Western blot were used to detect the expression of key genes in the mouse carotid artery. The findings revealed that the gene and protein expression levels of ATP2B1, CHST15, CSTB and RNASE1 in the ApoE^−/−^ +HFD group were higher than those in C57BL/6J mice. Among them, the expression levels of CHST15, CSTB and RNASE1 were significantly higher than those of C57BL/6J mice (*P* < 0.001) (Figure 7).

**FIGURE 8:**
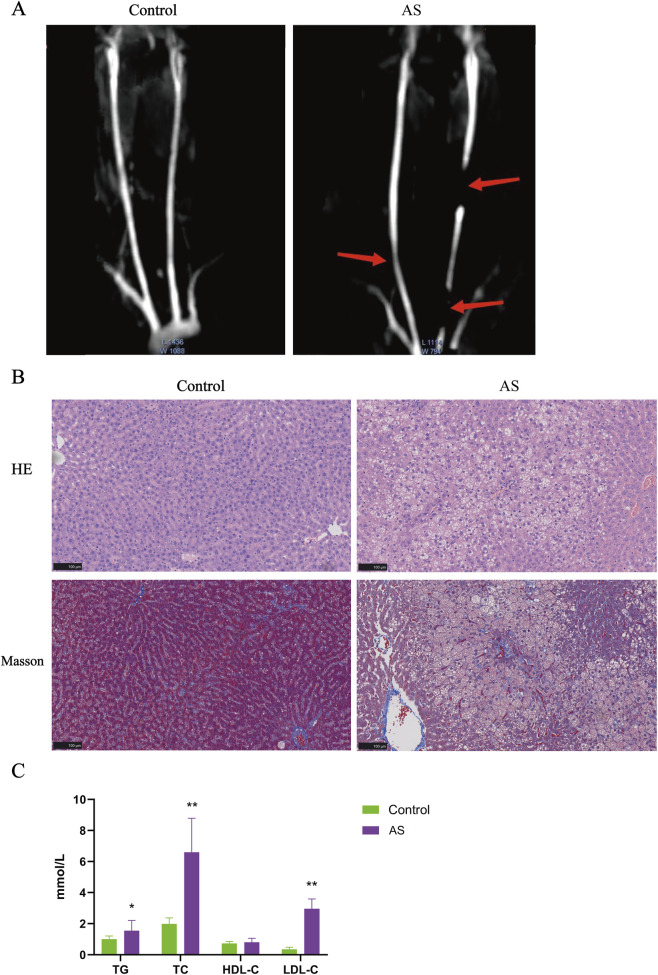
Validation of atherosclerosis model in rats. **(A)** Representative images of the common carotid artery examined by MRI (n = 4). **(B)** Representative images of liver stained with HE (top panel) and Masson (bottom panel) (n = 4). **(C)** Blood lipid level in rats (n = 4). Error bars represent standard deviation (*P < 0.05, **P < 0.01, compared with the Control group, two-tailed Student’s t-test).

**FIGURE 9:**
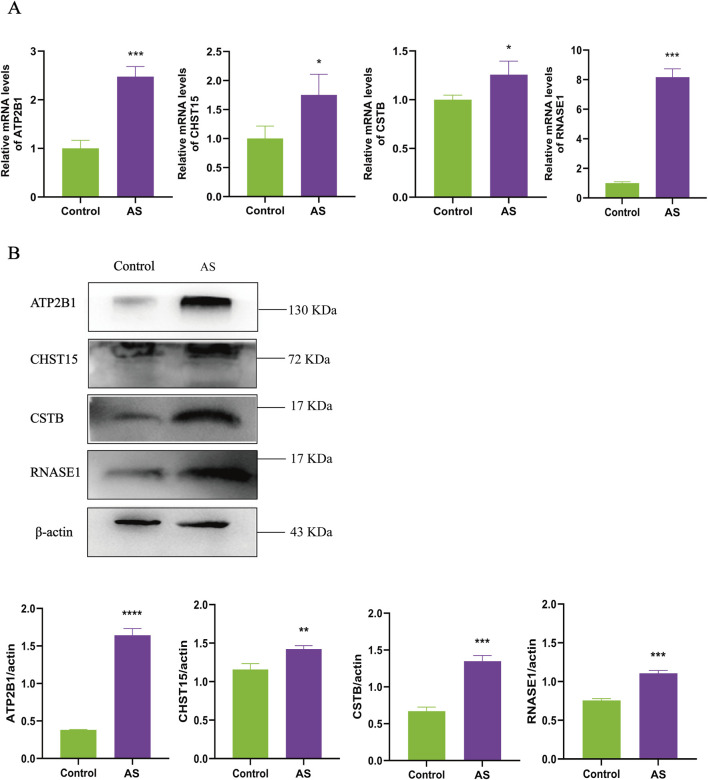
Expression levels of ATP2B1, CHST15, CSTB and RNASE1 in rat arteries. **(A)** The gene levels of ATP2B1, CHST15, CSTB and RNASE1 were analyzed by Real-time PCR. **(B)** The protein levels of ATP2B1, CHST15, CSTB and RNASE1 were analyzed by western blot. Actin was used as a loading control. Data represent mean ± standard deviation of three independent experiments (*P < 0.05, **P < 0.01, ***P < 0.001, compared with the Control group, two-tailed Student’s t-test).

This study employed a combined approach of “carotid artery endothelial injury + vitamin D3 injection + long-term high-fat diet” to induce atherosclerotic plaque formation in SD rats. MRI has long been esteemed as the gold standard for non-invasive assessment of carotid plaque morphology and composition (Lavin et al., 2021). The AS group exhibited stenosis or complete occlusion of the carotid arteries, suggesting the potential formation of plaque beneath the vessel intima (Figure 8A). HE staining or Masson staining revealed marked hepatocyte edema and the presence of cytoplasmic vacuoles in the livers of the AS group. Conversely, no notable pathological features were observed in the livers of the control group. These findings indicated the presence of moderate steatosis and mild infiltration of inflammatory cells in the livers of the AS group (Figure 8B). At the same time, we measured the levels of TG, TC, LDL-C, and HDL-C in the serum of rats. The results showed that the levels of TG, TC, and LDL-C in the AS group were significantly higher than those in the control group (*P* < 0.05), while the level of HDL-C did not change significantly (*P* > 0.05) (Figure 8C). These findings preliminary suggest that the AS group exhibits disrupted lipid metabolism, elevated liver lipid accumulation, and vascular abnormalities, leading to the development of atherosclerosis. Importantly, Real-time PCR and Western blot analyses revealed that the expression of ATP2B1, CHST15, CSTB, and RNASE1 was significantly upregulated to varying degrees in the carotid arteries of the AS group compared to the control group (*P* < 0.05) (Figure 9). Our *in vivo* studies provide further validation, demonstrating that the upregulation of ATP2B1, CHST15, CSTB, and RNASE1 is associated with atherosclerotic progression, thus strengthening the link between these markers and the disease.

### 3.6 Overview of key gene expression in single cell data and analysis of immune infiltration

**FIGURE 10:**
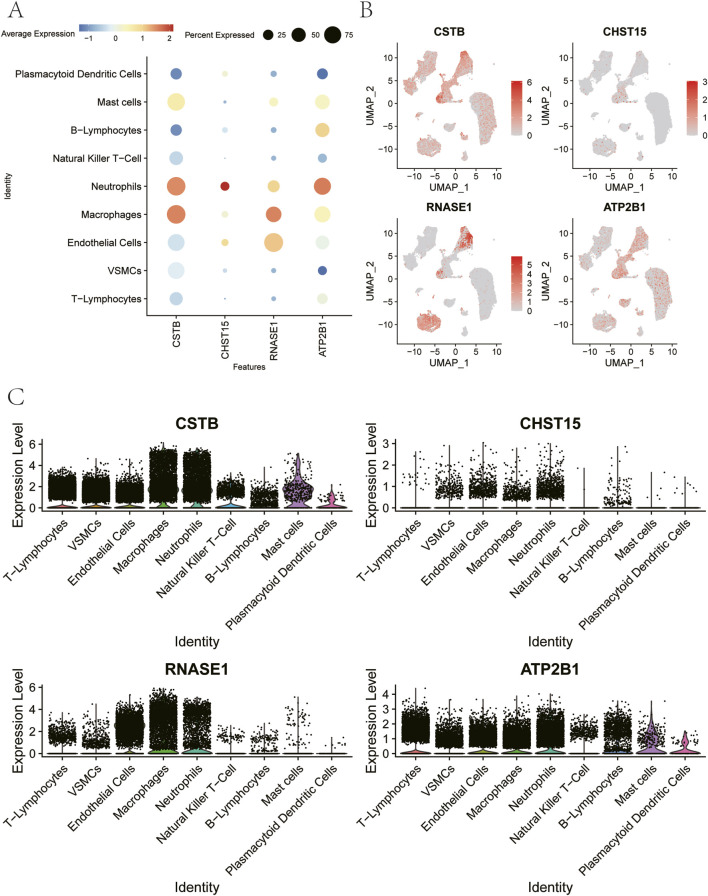
Expression levels of ATP2B1, CHST15, CSTB and RNASE1 in different cell subtypes. **(A)** Dot plot showing average expression (−1 to 2) of genes across immune cell types. **(B)** UMAP plots of percent expression for CSTB, CHST15, RNASE1, and ATP2B1 in single cells. **(C)** The expression level distribution of selected genes in different cell subtypes.

This study analyzed the key genes expression in single-celled, and shows the CSTB, CHST15, RNASE1, ATP2B1 in T-Lymphocytes, VSMCs, Endothelial Cells, Macrophages, Neutrophils, Expression of Natural Killer T-Cell, B-Lymphocytes, Mast cells, Plasmacytoid Dendritic Cells (Figure 10).

**FIGURE 11:**
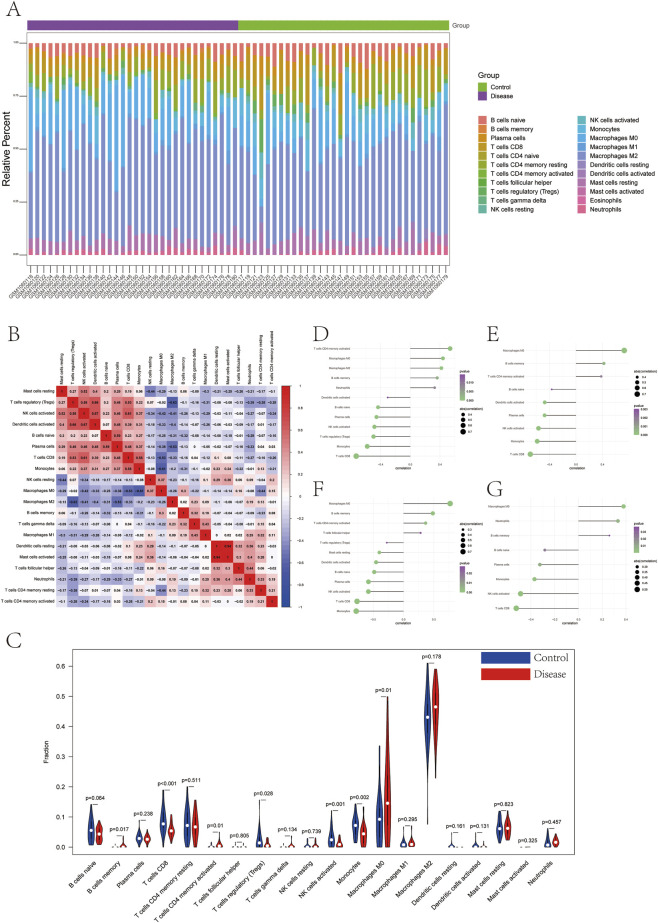
Infiltration of immune-associated cells in Control and Disease samples. **(A)** Relative percentage of 22 subpopulations of immune cells in each sample. **(B)** Interaction analysis among 22 different immune cells in disease group. **(C)** Differences in immune cell infiltration between Control and Disease groups. **(D)** The correlation between ATP2B1 and immune cells. **(E)** The correlation between CHST15 and immune cells. **(F)** The correlation between CSTB and immune cells. **(G)** The correlation between RNASE1 and immune cells.

We investigated the association between the key genes and immune infiltration to elucidate their role in atherosclerosis. The results delineated the immune landscape, including the specific proportion of various immune cells in each patient and the significant correlations observed between different immune cell types. (Figure 11A, B). Furthermore, the proportion of M0 macrophages was significantly higher in patients of the disease group than in those of the control group (Figure 11C).

We further explored the relationship between key genes and immune cells, and found that key genes were highly correlated with immune cells. ATP2B1 is significantly positively correlated with T cells CD4 memory activated, and significantly negatively correlated with T cells CD8 (Figure 11D). CHST15 is positively correlated with Macrophages M0 and negatively correlated with T cells CD8 (Figure 11E). CSTB is positively correlated with Macrophages M0, and negatively correlated with Monocytes (Figure 11F). RNASE1 is positively correlated with Macrophages M0 and negatively correlated with T cells CD8 (Figure 11G).

**FIGURE 12:**
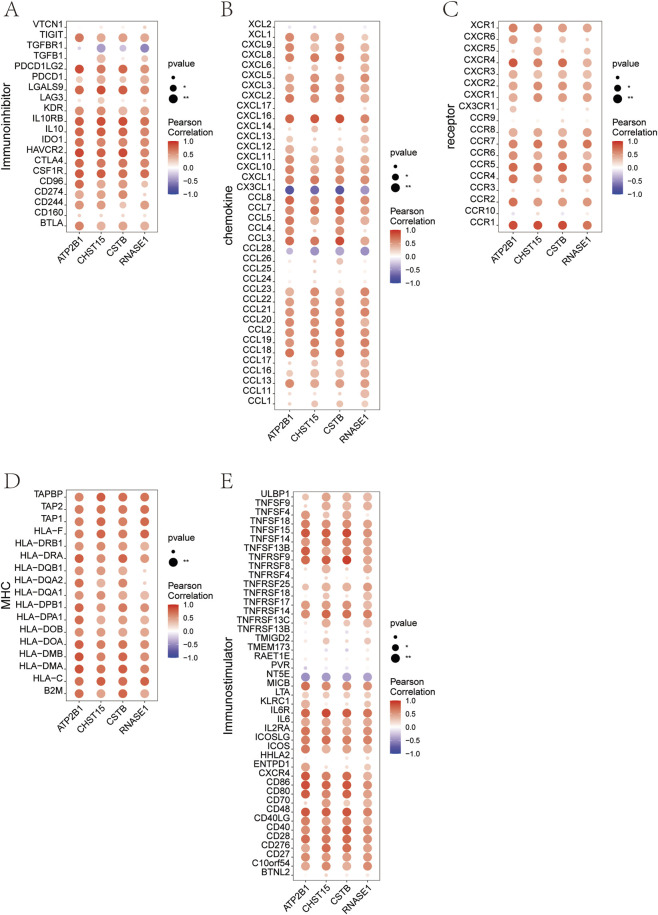
The correlation between four key genes and different immune factors. **(A)** Pearson correlation coefficients between expression of four genes and immunoinhibitor genes. **(B)** Pearson correlation coefficients between expression of four genes and chemokine genes. **(C)** Pearson correlation coefficients between expression of four genes and receptor genes. **(D)** Pearson correlation coefficients between expression of four genes and MHC genes. **(E)** Pearson correlation coefficients between expression of four genes and immunostimulator genes. Dot color indicates correlation value (red: positive, blue: negative, scale −1 to 1); dot size represents statistical significance (p value).

This study analyzed the correlation between four key genes and different immune factors, including immunosuppressive factors, immunostimulators, chemokines, and receptors. These analyses suggest that key genes are closely related to immune cell infiltration levels and play an important role in the immune microenvironment (Figure 12).

### 3.7 Signaling pathways involved in key genes

**FIGURE 13:**
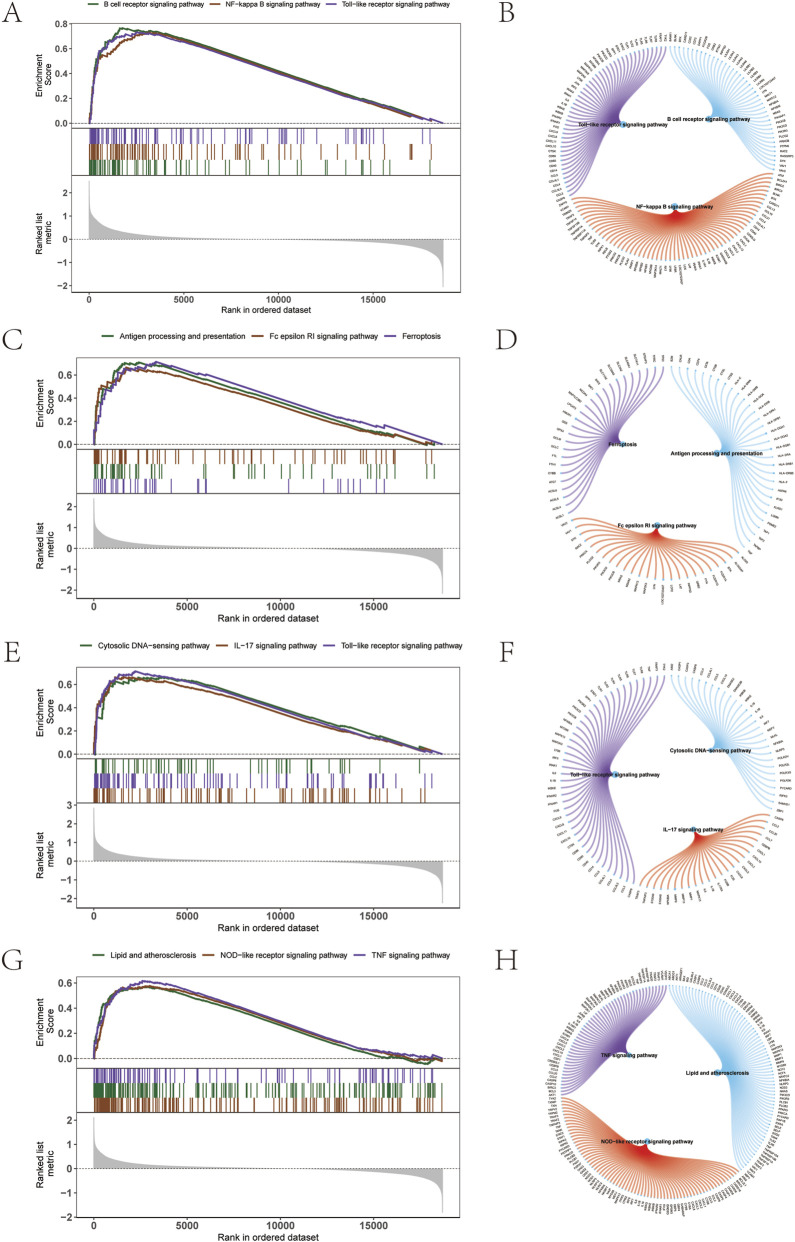
GSEA enrichment analysis of key genes in atherosclerotic disease. **(A, B)** GSEA of ATP2B1. **(C, D)**: GSEA of CHST15. **(E, F)** GSEA of CSTB. **(G, H)** GSEA of RNASE1.

GSEA results showed that, ATP2B1 was significantly enriched in B cell receptor signaling pathway, NF−kappa B signaling pathway, and Toll−like receptor signaling pathway (Figure 13A, B); CHST15 was significantly enriched in Antigen processing and presentation, Fc epsilon RI signaling pathway, Ferroptosis (Figure 13C, D). CSTB was significantly enriched in Cytosolic DNA−sensing pathway, IL−17 signaling pathway, and Toll−like receptor signaling pathway (Figure 13E, F). RNASE1 was significantly enriched in Lipid and atherosclerosis, NOD−like receptor signaling pathway, and TNF signaling pathway (Figure 13G, H).

**FIGURE 14:**
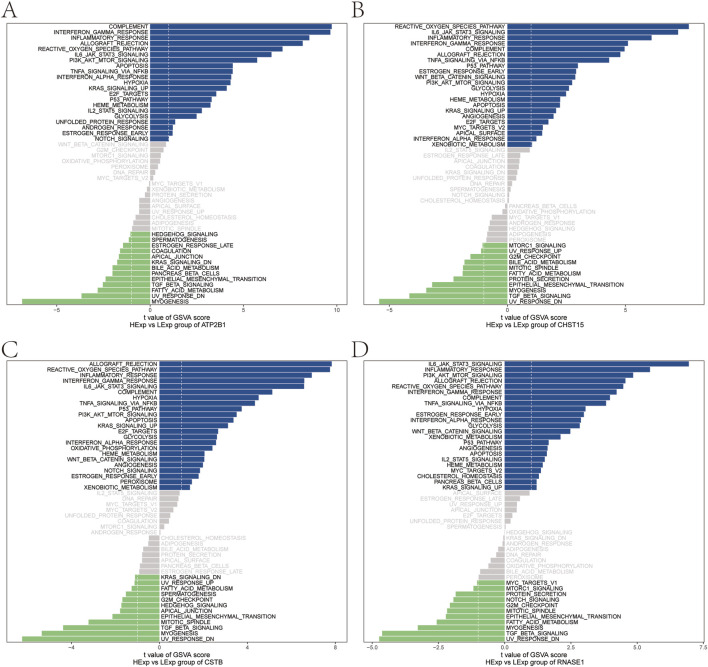
GSVA enrichment analysis of key genes in atherosclerotic disease. **(A)** GSVA of ATP2B1. **(B)** GSVA of CHST15. **(C)** GSVA of CSTB. **(D)** GSVA of RNASE1.

GSVA results showed that high expression of ATP2B1 was enriched in signaling pathways such as COMPLEMENT and INTERFERON GAMMA RESPONSE (Figure 14A). CHST15 was prominently enriched in REACTIVE OXYGEN SPECIES PATHWAY, IL6 JAK STAT3 SIGNALING, and INFLAMMATORY RESPONSE (Figure 14B). CSTB was prominently enriched in ALLOGRAFT REJECTION, REACTIVE OXYGEN SPECIES PATHWAY, and INFLAMMATORY RESPONSE (Figure 14C). Highly expressed RNASE1 was enriched in IL6 JAK STAT3 SIGNALING, INFLAMMATORY RESPONSE, PI3K AKT MTOR SIGNALING (Figure 14D). This suggests that key genes may influence the progression of atherosclerosis through these pathways.

### 3.8 Transcriptional regulation analysis of key genes

**FIGURE 15:**
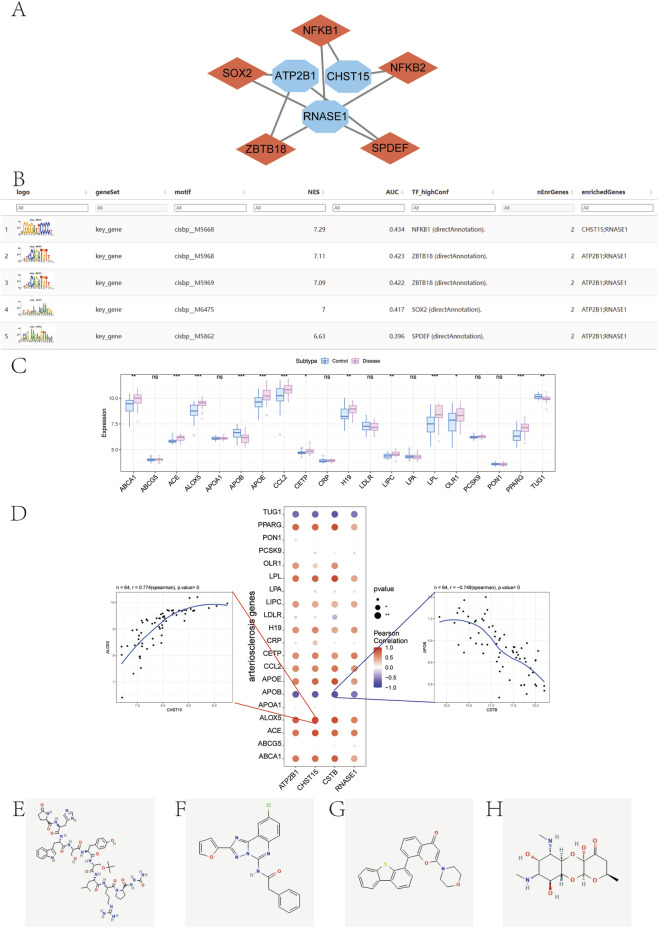
**(A, B)**: Motif transcriptional regulation analysis. **(A)** Demonstration of the highest motif enrichment of AUC, including NES (standardized enrichment score for motifs in gene sets), AUC (area under the curve), and TF_highConf (transcription factors annotated to motifs). **(B)** miRNA networks of key genes, blue for mRNA and red for miRNA. **(C)** Expression of disease-regulating genes in control group and disease group. **(D)** Correlation between key genes and disease-regulating genes. **(E–H)** Molecular structure diagram of NU-7441, Goserelin, Spectinomycin, and MRS-1220.

To explore potential shared regulatory mechanisms, we performed a transcription factor (TF) binding motif enrichment analysis on the promoter regions of the four key genes (ATP2B1, CHST15, CSTB, and RNASE1). The analysis revealed that these genes possess common overrepresented cis-regulatory motifs, suggesting co-regulation by a set of specific TFs. The most significantly enriched motif was identified as cisbp__M5668 (normalized enrichment score, NES = 7.29). All enriched motifs and their corresponding predicted transcription factors were showed in Figure 15A, B. This finding indicates that the coordinated upregulation of these genes in atherosclerosis may be driven by a common transcriptional regulatory program.

### 3.9 Correlation between key genes and disease-regulating genes

To validate the disease relevance of the identified key genes, we examined their relationship with a curated set of atherosclerosis-regulatory genes obtained from the GeneCards database (https://www.genecards.org/). First, we confirmed that this disease gene set was differentially expressed between our patient cohorts. Genes such as ABCA1, ALOX5, and ACE showed significant expression differences (*P* < 0.05), confirming the validity of our clinical grouping (Figure 15C). Subsequently, correlation analysis revealed that the expression levels of our four key genes were significantly associated with several core disease regulators. Specifically, CHST15 and ALOX5 were significantly positively correlated (*r* = 0.774), and CSTB and APOB were significantly negatively correlated (*r* = −0.748) (Figure 15D).

### 3.10 Cmap drug prediction

In this study, limma package was used to calculate differential genes among patients with GSE43292 expression profile. The screening criteria for differential genes were logFC >0.585 and *P*. Vue <0.05. A total of 913 differential genes were finally screened, including 531 upregulated genes and 382 downregulated genes. We downregulated the differential mRNA of Top 150 groups and used the Connectivity Map database to predict drug targeting of differential genes. The results showed that the expression profiles of interferents such as NU-7441, Goserelin, Spectinomycin, and MS-1220 are significantly negatively correlated with those of disease-interfering substances (Figures 15E–H). This drug may be able to alleviate and even reverse the disease state. Future studies are required to test these compounds in animal models of atherosclerosis to validate their efficacy and elucidate their mechanisms of action.

## 4 Discussion

This study provides novel insights into atherosclerosis pathogenesis through comprehensive single-cell transcriptomic analysis. Our findings contribute to the field in three major aspects: (1) detailed characterization of neutrophil heterogeneity in atherosclerotic lesions, (2) the four identified key genes show potential as diagnostic biomarkers, and (3) elucidation of possible molecular pathways underlying atherosclerosis progression. By integrating scRNA-seq data from multiple human atherosclerotic samples, we identified four key genes within neutrophil subtypes-CSTB, CHST15, RNASE1, and ATP2B1 - as potential biomarkers for the progression of atherosclerosis, especially for plaque instability, and may provide a theoretical basis for the development of new therapies targeting neutrophil inflammation.

Our findings reinforce several established concepts in atherosclerosis research. First, we observed a significantly higher proportion of neutrophils in diseased arteries compared to controls, consistent with previous studies demonstrating the crucial role of neutrophils in atherogenesis (Matthias Nahrendorf, 2015). The identification of distinct neutrophil subpopulations aligns with recent single-cell studies showing immune cell heterogeneity in atherosclerotic plaques (Yuan et al., 2024). Furthermore, our pathway analysis confirms the central role of inflammatory responses and immune cell interactions in disease progression, supporting current understanding of atherosclerosis pathophysiology.

The integration of these four genes into a diagnostic signature represents an innovative approach, moving beyond single-marker strategies commonly used in cardiovascular diagnostics. Most notably, we uncovered previously unrecognized roles of CSTB and CHST15 in atherosclerosis. CSTB, an endogenous inhibitor of lysosomal cysteine proteases, has been shown to be upregulated in ovarian, liver, and colon cancers (Wang et al., 2014). Cystatin B, encoded by CSTB, has been proposed as a cardiovascular disease biomarker in plasma (Darmanis et al., 2011; Gonçalves et al., 2016). While CSTB has been primarily studied in cancer progression, our results reveal its unexpected negative correlation with APOB in atherosclerotic lesions, suggesting a novel regulatory mechanism in lipid metabolism. Similarly, CHST15, known for its involvement in chronic inflammation (Suzuki et al., 2016), showed a strong positive correlation with ALOX5, identifying a potential new axis in the regulation of oxidative stress and ferroptosis in atherosclerosis.

RNASE1, an enzyme essential for maintaining vascular homeostasis, protects endothelial cells from extracellular RNA damage during acute inflammation, contributing to vascular health (Bedenbender and Schmeck, 2020). Notably, recent studies have identified RNASE1 as a potential atherosclerosis biomarker, with RNASE1 knockdown reducing macrophage migration, lowering total and LDL cholesterol levels, increasing HDL cholesterol levels, and significantly decreasing atherosclerotic plaque formation (Chen et al., 2024). Furthermore, ATP2B1, encoding plasma membrane calcium ATPase 1 (PMCA1), plays a crucial role in maintaining intracellular calcium homeostasis. Its knockout leads to increased intracellular calcium concentrations, affecting vascular smooth muscle cell contractility and resulting in elevated blood pressure (Kobayashi et al., 2012). However, ATP2B1 overexpression may also cause excessive calcium efflux, impacting normal physiological functions of endothelial cells and other cell types. These studies further support the reliability of our findings.

Our study also explores the potential molecular mechanisms and signaling pathways through which the key genes influence atherosclerosis progression. Immune infiltration analysis revealed a significant increase in macrophages M0 within the disease group compared to healthy controls. Among them, CHST15, CSTB, and RNASE1 showing significant positive correlations with macrophages M0. Previous studies have shown that the majority of immune cells in early and late plaques are M0 and M2 macrophages, CD4 memory T cells, and CD8 T cells (Gao et al., 2021), consistent with our findings. CHST15, CSTB, and RNASE1 may regulate the polarization of M0 macrophages into pro-inflammatory M1 or anti-inflammatory M2 types.

Importantly, GSEA analysis revealed that the signaling pathways enriched for the key genes are primarily related to immune signal transduction, as well as inflammation-associated pathways such as IL-17, NOD-like receptor, and TNF signaling, and processes like ferroptosis and lipid metabolism in atherosclerosis. The NF-κB, Toll-like receptor, IL-17, NOD-like receptor, and TNF signaling pathways, significantly enriched for ATP2B1, CSTB, and RNASE1, have been recognized to directly influence the pathophysiology of atherosclerosis by regulating inflammatory responses, immune responses, endothelial dysfunction, and smooth muscle cell proliferation (Marion Mussbacher, 2019; Jin et al., 2023; Moeinafshar et al., 2022; Williams et al., 2019). The Fc epsilon RI signaling pathway, significantly enriched for CHST15, promotes atherosclerosis development by mediating IgE-induced immune responses and vascular remodeling (Wang et al., 2011). Ferroptosis, a novel form of cell death, plays a significant role in atherosclerosis by affecting the death and survival of vascular wall cells and pathological processes such as oxidative stress (Lin et al., 2022).

Furthermore, GSVA analysis showed that pathways related to immune response, inflammatory response, and oxidative stress were also activated in samples with high expression of the key genes. Inflammatory responses play a crucial role in atherosclerosis progression, potentially impacting initial leukocyte recruitment up to the rupture of unstable atherosclerotic plaques (Fragoso-Lona et al., 2009). In atherosclerotic samples, high CHST15 expression was enriched in the IL-6/JAK/STAT3 signaling pathway. Recinos et al. found that Angiotensin II activates the Jak-STAT3 signaling pathway by inducing IL-6 production in adventitial fibroblasts and activated macrophages in the aorta, playing a key role in the development of early atherosclerotic lesions (Recinos et al., 2007). Similarly, in myeloproliferative neoplasm samples, high CHST15 expression may participate in cell proliferation and differentiation through the IL-/JAK/STAT3 signaling pathway (Chen et al., 2022). High CSTB expression was enriched in the ALLOGRAFT REJECTION signaling pathway. Previous studies have shown that allograft vascular disease is a special case of immune-mediated atherosclerosis (Libby, 2012). These results support the potential of the four key genes as therapeutic targets for atherosclerosis, indicating the regulatory pathways leading to vascular inflammation and atherosclerosis development, and facilitating the development of novel therapies to control atherosclerosis progression.

Currently, effective pharmacological treatments for atherosclerosis are still lacking, and there is an urgent need to explore potential drugs. However, previous studies have not used high-throughput screening based on gene expression profile characteristics to reveal potential small molecule compounds for atherosclerosis treatment. In this study, we applied differentially expressed genes associated with atherosclerosis to cMAP analysis and selected four small molecule compounds (NU-7441, Goserelin, Spectinomycin, MRS-1220) with high negative enrichment scores as candidate compounds. In the treatment of uterine leiomyoma, Goserelin has been observed to reduce the levels of inflammatory factors such as TNF-α and MCP-1 in patients, suggesting that Goserelin may alleviate inflammatory1 responses (Mohammed et al., 2020). MRS-1220 has shown significant anti-angiogenic effects in both *in vivo* and *in vitro* studies. In animal models, MRS-1220 has been proven to reduce tumor size and angiogenesis in glioblastoma (Rocha et al., 2018). In the human macrophage U-937 cell line, MRS-1220 can reverse the inhibition of tumor necrosis factor-α (TNF-α) formation caused by A3 agonists, indicating its potential role in regulating immune responses and inflammatory processes (Jacobson et al., 1997). As angiogenesis is involved in the atherosclerotic process, the anti-angiogenic effects of MRS-1220 may impact atherosclerosis progression to some extent. However, these drugs and targets are only theoretical predictions, and require further validation through animal experiments and clinical trials.

Despite these promising findings, several limitations of our study should be acknowledged. First, while our animal models validated the expression changes of key genes, these models may not fully recapitulate the complexity of human atherosclerosis. The ApoE^−/−^ mouse model and rat model, although widely used, have inherent limitations in mimicking human pathology, particularly regarding plaque stability and rupture characteristics. Second, while our diagnostic model showed promising results in both training and validation cohorts, these cohorts were retrospectively analyzed. Prospective validation in larger, more diverse patient populations is necessary to establish the clinical utility of our four-gene signature. Additionally, the influence of common cardiovascular risk factors and concurrent medications on these biomarkers needs to be evaluated. Third, our study’s focus on neutrophil populations, while providing valuable insights, may have overlooked important interactions with other cell types in the atherosclerotic microenvironment. A more comprehensive analysis of cell-cell interactions and their temporal dynamics during disease progression would enhance our understanding of the complex pathophysiology of atherosclerosis.

## 5 Conclusion

This study revealed the association between neutrophils and the progression of atherosclerosis, identified ATP2B1, CHST15, CSTB, and RNASE1 as the key regulatory factors in this network, and preliminarily explored the related molecular pathways. These genes have a positive or negative correlation with certain immune cells and may affect the immune microenvironment of atherosclerosis. Due to the insufficient sample size in this study and the potential limitations of the selected public data sets in representing a broader population of atherosclerosis, future functional studies are needed in appropriate models and prospective clinical cohorts to rigorously validate the role of key genes as diagnostic markers for unstable plaques. This study initially verified the high expression of key genes in diseased carotid artery tissues using animal models. The future work will focus on elucidating the expression levels and functional roles of key genes in the neutrophil population within the atherosclerotic microenvironment. Additionally, although we identified significant correlations between key genes and the expression levels of regulatory genes related to atherosclerosis such as ALOX5 and APOB, the mechanisms by which they interact to affect the progression of atherosclerosis still require further investigation. In summary, our study provides new insights into the diagnosis and treatment of unstable plaques in atherosclerosis and offers a new perspective for exploring the related genes and molecular mechanisms of atherosclerosis progression.

## Data Availability

The data supporting this study are publicly available from the NCBI GEO database under accession numbers GSE159677, GSE43292, and GSE28829.
