# Supplementary figures and images for: Single-cell transcriptomics reveals heterogeneous neutrophil populations and diagnostic biomarkers in atherosclerosis
Source: Front Physiol. 2026 Mar 2;17:1704443. doi: 10.3389/fphys.2026.1704443 (PMC12989409; doi:10.3389/fphys.2026.1704443)

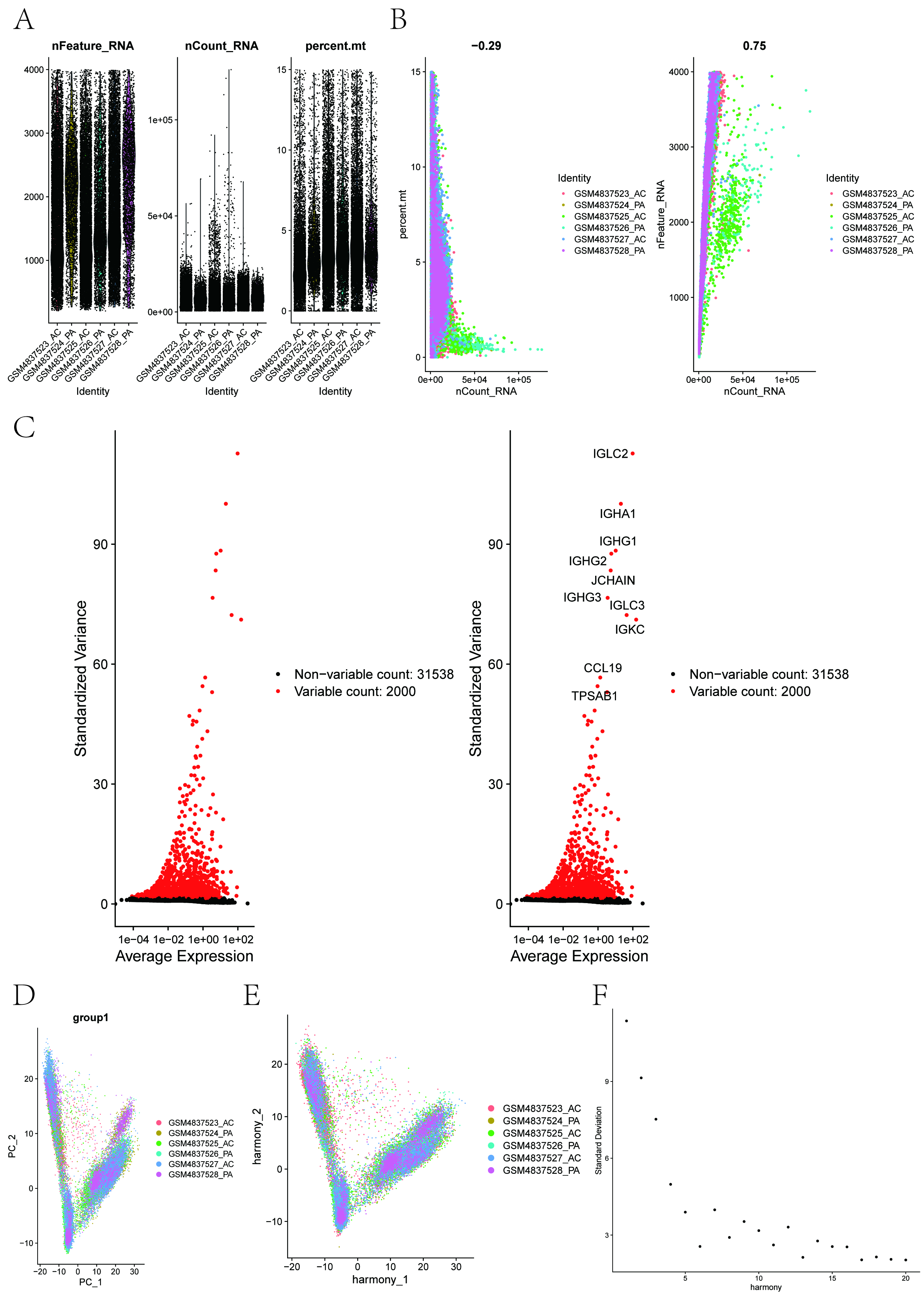

Supplement: Supplementary file 1 [file Image1.TIF]
